# AFM Study of Roughness Development during ToF-SIMS Depth Profiling of Multilayers with a Cs^+^ Ion Beam in a H_2_ Atmosphere

**DOI:** 10.1021/acs.langmuir.2c01837

**Published:** 2022-10-14

**Authors:** Jernej Ekar, Janez Kovač

**Affiliations:** † Jožef Stefan Institute, Jamova Cesta 39, SI-1000 Ljubljana, Slovenia; ‡ Jožef Stefan International Postgraduate School, Jamova Cesta 39, SI-1000 Ljubljana, Slovenia

## Abstract

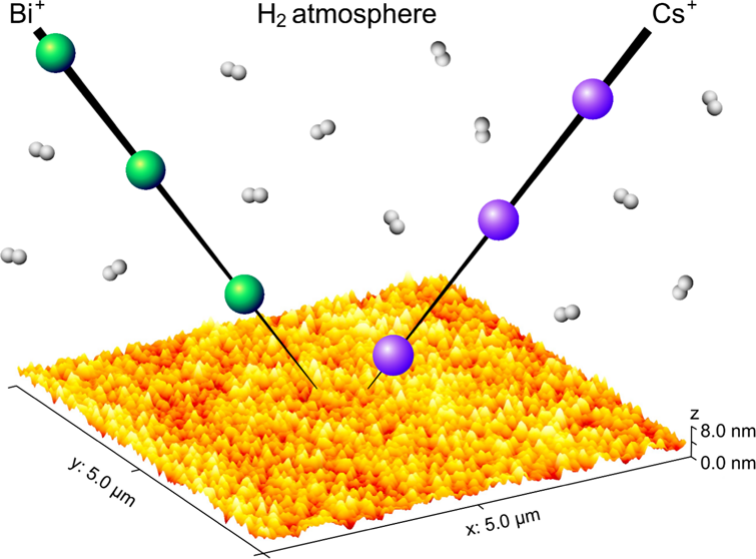

The influence of H_2_ flooding on the development of surface roughness during time-of-flight secondary ion mass spectrometry (ToF-SIMS) depth profiling was studied to evaluate the different aspects of a H_2_ atmosphere in comparison to an ultrahigh vacuum (UHV) environment. Multilayer samples, consisting of different combinations of metal, metal oxide, and alloy layers of different elements, were bombarded with 1 and 2 keV Cs^+^ ion beams in UHV and a H_2_ atmosphere of 7 × 10^–7^ mbar. The surface roughness *S*_a_ was measured with atomic force microscopy (AFM) on the initial surface and in the craters formed while sputtering, either in the middle of the layers or at the interfaces. We found that the roughness after Cs^+^ sputtering depends on the chemical composition/structure of the individual layers, and it increases with the sputtering depth. However, the increase in the roughness was, in specific cases, approximately a few tens of percent lower when sputtering in the H_2_ atmosphere compared to the UHV. In the other cases, the average surface roughness was generally still lower when H_2_ flooding was applied, but the differences were statistically insignificant. Additionally, we observed that for the initially rough surfaces with an *S*_a_ of about 5 nm, sputtering with the 1 keV Cs^+^ beam might have a smoothing effect, thereby reducing the initial roughness. Our observations also indicate that Cs^+^ sputtering with ion energies of 1 and 2 keV has a similar effect on roughness development, except for the cases with initially very smooth samples. The results show the beneficial effect of H_2_ flooding on surface roughness development during the ToF-SIMS depth profiling in addition to a reduction of the matrix effect and an improved identification of thin layers.

## Introduction

Ion sputtering is the main process taking place during an analysis based on secondary ion mass spectrometry (SIMS).^1^ It is also an essential process for depth profiling, combined with X-ray photoelectron spectroscopy (XPS) and Auger electron spectroscopy (AES).^2−4^ All three methods employ ion guns for sputtering, although the sputtering process itself is also present in the case of glow-discharge optical emission spectroscopy (GDOES) or glow-discharge mass spectrometry (GDMS).^3,5−7^ The main difference is the characteristic of the GDOES or GDMS processes, as ions are intrinsic to the plasma that flows toward the cathode, causing its surface to be sputtered away.^6,8^ These methods are used in many areas of research, for example, during the analysis of oxide layers,^9^ while studying corrosion properties,^10^ polymer films,^11^ mono- and multilayers,^12^ biomaterials,^13^ microelectronics,^14,15^ power-storage materials,^16^ solar cells,^17,18^ and catalysts.^19^ However, regardless of the exact process used for the ion sputtering, the ion beam generated by the ion gun or the plasma flow, some damage caused by the ion bombardment is always present.^20−22^ The accumulation of damage is observed as surface roughening, which is most commonly determined with atomic force microscopy (AFM).^23,24^ This technique is especially suitable for the characterization of nanostructures formed on the surface since it is optimized to achieve molecular and atomic resolutions.^25−27^ However, AFM can also be used to study many other topographical characteristics of the sample, its conductivity at the nano level, and different forces using its spectroscopy mode.^27−31^

The general behavior related to sputter-induced damage and surface roughening is that sputtering ions with a higher energy^21,32,33^ and a longer sputtering process^21,33−35^ lead to a greater surface roughness being caused by the ions. Since surface roughening is an unwanted process in many applications, different approaches have been developed to reduce it as much as possible. Many investigations have looked at temperature manipulation, the type and size of the sputtering ions as well as adjusting the angle at which they bombard the surface, the use of sample rotation,^36,37^ and finally gas flooding.^38,39^ It was shown that the depth profiles of mainly polymeric, organic, and biological, but also inorganic compounds can be improved, as well as the sputter-induced topography reduced, if the sample is cooled.^40−42^ The depth profiling of organic materials was also improved by the application of larger, molecular sputter ions such as Bi_3_^+^, Au_3_^+^, SF_5_^+^, C_60_^+^ (fullerene), and Ar_*n*_^+^ (argon clusters with *n* being the number of Ar atoms, which is between a few hundred and a few thousand).^37,43,44^ Furthermore, we can improve the depth resolution by bombarding the sample with ions at larger incident angles with respect to the surface normal (grazing angles).^45,46^ But regardless of the angle of bombardment, topographical structures such as ripples, ridges, valleys, cones, and pyramids are formed during the sputtering process.^35,40,47^ Their formation can be suppressed and the surface roughening reduced by sample rotation.^22,48,49^ Last but not least, it was also shown that surface roughening can be reduced if an O_2_ atmosphere is applied during the depth profiling, causing the sample to oxidize.^38,39^

As we have shown in our recent work, the time-of-flight secondary ion mass spectrometry (ToF-SIMS) depth profiling of metals, metal oxides, and alloys is improved in terms of sensitivity and a reduction of the matrix effect if a H_2_ atmosphere is applied instead of ultrahigh vacuum (UHV).^12^ Since H_2_ flooding is a novelty in the field of atmosphere manipulation and has, at least to some degree, similar effects to O_2_ flooding, we made AFM measurements of the sputtered craters and checked whether less surface roughening can be observed in the case of our experiments as well. In this study, we depth-profiled four different samples composed of metal, metal oxide, and alloy multilayers while sputtering with a Cs^+^ ion beam in both UHV and H_2_ environments. Furthermore, we tested different energies of Cs^+^ sputtering ions and analyzed the surface roughness of the layers with different chemical compositions. Last but not least, the samples analyzed had different initial surface roughnesses, another factor that influenced the surface morphology during sputtering. We showed that a H_2_ atmosphere generally reduces the surface roughening or leads to no statistically significant change.

## Experimental Section

### Preparation of the Samples

All of the metals and metal oxides were prepared using physical vapor deposition (PVD). They were deposited in a Sputron triode sputtering system (Balzers Oerlikon). The background pressure was lower than 1 × 10^–6^ mbar. The partial pressure of the argon working gas in the vacuum chamber was 2 × 10^–3^ mbar for all of the processes. A maximum substrate temperature of less than 100 °C was maintained during the deposition. A quartz-crystal microbalance was used to calibrate the deposition rates. The deposition rates and thickness reproducibility were better than 2%.

The 60 mm diameter targets were initially cleaned for 5 min to remove the native oxide and other impurities on their surfaces. High-purity targets were used as the sputtering source. Metal-oxide layers (Cr_2_O_3_, TiO_2_, Al_2_O_3_, Fe_2_O_3_, NiO) were prepared by reactive sputtering. In this process, thin oxide films were deposited on the substrates by sputtering metallic targets in the presence of oxygen mixed with an argon working gas. The flow rate of the oxygen (99.998%) was controlled with a flowmeter.

### Ion Sputtering of the Samples

Samples were sputtered using Cs^+^ ions with energies of 1 and 2 keV and ion currents of 49–67 and 78–90 nA, respectively. The 1 keV Cs^+^ was sputtered in pulses lasting 48.5 μs, while the 2 keV Cs^+^ used pulses of 61.5 μs. Pulsed Bi^+^ primary ions with an energy of 30 keV, a pulse length of 5.9 ns, and an ion current of 0.8–2.2 pA were used for the analysis. The Cs^+^ and Bi^+^ ions were generated in two separate ion guns (dual-beam depth profiling) mounted on a TOF.SIMS 5 instrument produced by IONTOF GmbH (Munster, Germany). The ion guns work interchangeably and sequentially. Namely, we have a cycle of ion-etching with the Cs^+^ ions, which happens while the separation in the time-of-flight analyzer and detection takes place (70 μs). The Cs^+^ cycle is shorter than the ToF analysis, so we also have a time interval without any sputtering. This is followed by the cycle of ion sputtering with the Bi^+^ ions, being the basis for the next ToF analysis. Sputtering with the Bi^+^ primary ions was performed over a 200 μm × 200 μm scanning area (128 pixels × 128 pixels), located in the center of the 400 μm × 400 μm etching crater created by the Cs^+^ ion beam.

The H_2_ used during the depth profiling was introduced into the analysis chamber close to the analyzed region (a distance of less than 1 cm). The gas introduction was manually controlled with a precise gas-leak valve through a capillary, leading toward the analyzed area. H_2_ with a 6.0 purity was used and the pressure inside the analysis chamber during the gas flooding was approximately 7 × 10^–7^ mbar. Analyses in the UHV conditions were made in the pressure range between 6 × 10^–10^ and 4 × 10^–9^ mbar.

### AFM Measurements

Surface roughness was determined with a Solver PRO 47 AFM microscope produced by NT-MDT (Russia) with AFM tips produced by the same company. The arithmetic average of the three-dimensional (3D) roughness (*S*_a_) was chosen as the representative value. Images were measured on 2 μm × 2 μm and 5 μm × 5 μm scanning areas in semicontact mode. The recording frequency was 1.0 Hz. The resolution of the images was set to 256 pixels × 256 pixels. The plane subtraction of the AFM images due to the inclination of the samples involved a second-order polynomial correction.

## Results and Discussion

### Composition of the Samples

**Figure 1:**
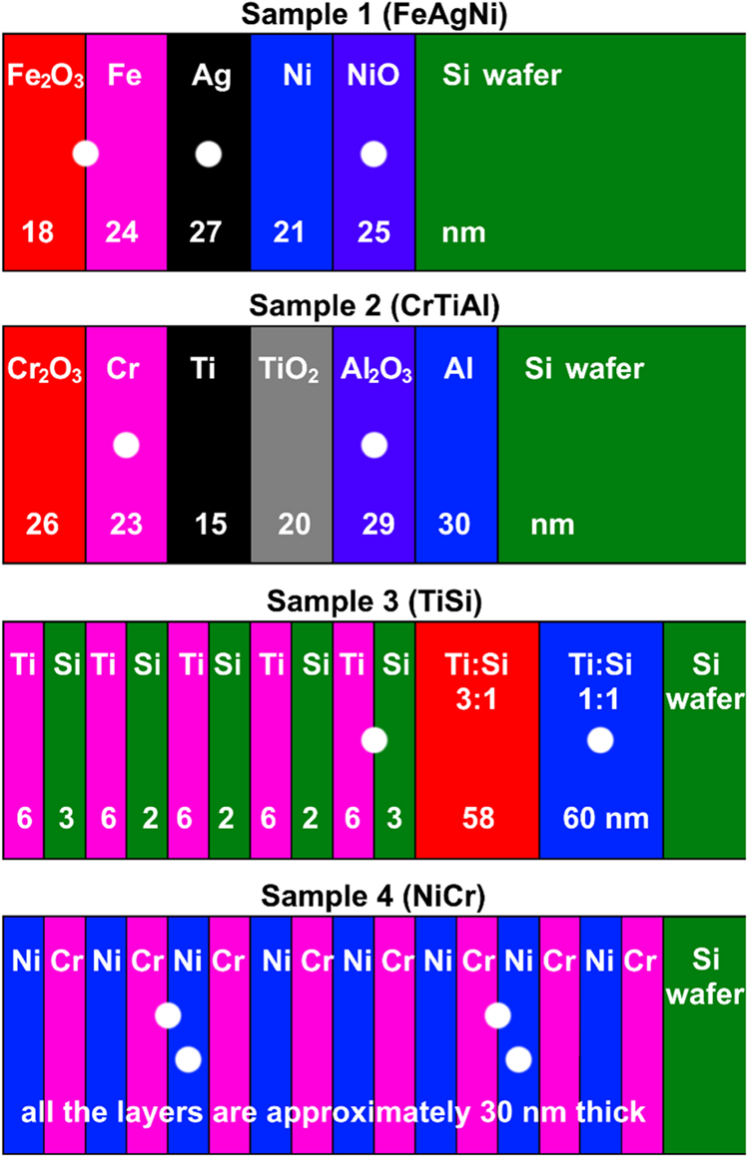
Schematic of four samples with layer thicknesses. White dots represent interfaces and layers where the analyses of the surface roughnesses were made. Adapted with permission from ref (12). Copyright 2022 creative commons.

Four different samples with multilayer structures of metals, metal oxides, and alloys were analyzed. All of the samples were prepared on mirror-like polished silicon wafers and had a different initial surface roughness. Sample 1 (FeAgNi) and sample 2 (CrTiAl) were composed of Fe_2_O_3_/Fe/Ag/Ni/NiO and Cr_2_O_3_/Cr/Ti/TiO_2_/Al_2_O_3_/Al layers, respectively. Sample 3 (TiSi) consisted of 10 alternating layers of Ti and Si followed by two Ti–Si alloy layers. The first layer had a stoichiometric ratio of Ti and Si equal to 3:1, with the higher concentration being titanium, while the second layer had a Ti/Si ratio of 1:1. Sample 4 (NiCr) consisted of 16 alternating layers of Ni and Cr. The exact structure of each sample is shown in Figure 1, together with the thickness of each layer. The surface roughness was measured on the initial nonsputtered surface of all four samples, at different interfaces, and in the middle of the chosen layers. The analyzed interfaces were:

- Fe_2_O_3_/Fe interface (FeAgNi sample)
- 5th Ti/Si interface (TiSi sample)
- 2nd and 6th Cr/Ni interface (NiCr sample)

The layers for which we analyzed the surface roughness were:

- Ag and NiO (FeAgNi sample)
- Cr and Al_2_O_3_ (CrTiAl sample)
- Ti–Si alloy with the atomic ratio of 1:1 (TiSi sample)
- 3rd and 7th Ni layer (NiCr sample)

The depths where the analyses were made are also indicated in Figure 1 as white circles at the interfaces and in the middle of the layers.

### SIMS Depth Profiles

**Figure 2:**
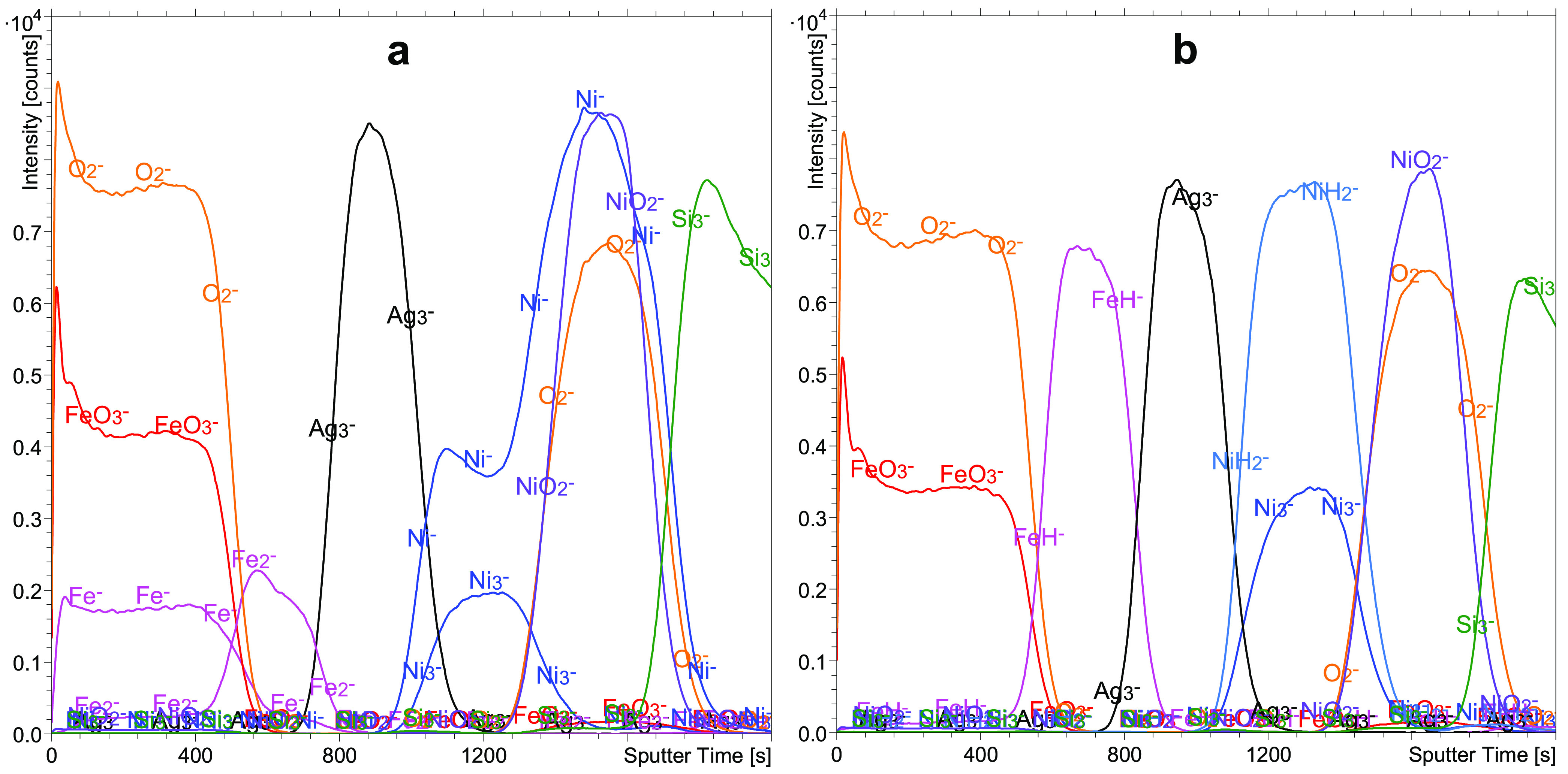
Depth profiles of the FeAgNi sample recorded using a 1 keV Cs^+^ sputtering beam. Profile (a) was recorded in a UHV environment, while profile (b) was recorded during H_2_ flooding. The presence of H_2_ is the reason for the intense metal hydride signals in profile (b). The intensities of some signals were multiplied by a specific factor as a way of reducing the intensity scale interval and making the profile clearly readable. Adapted with permission from ref (12). Copyright 2022 creative commons.

Figure 2a shows the ToF-SIMS depth profile of the FeAgNi sample recorded in UHV conditions, and Figure 2b shows the depth profile of the same sample recorded during H_2_ flooding. The signals of the different secondary ions are shown as a function of the sputtering time. In the ToF-SIMS depth profile shown in Figure 2a, different metal and metal oxide layers cannot be clearly identified. On the other hand, the depth profile recorded during H_2_ flooding (Figure 2b) unambiguously describes the exact structure of the FeAgNi sample. The roughness developed during ion sputtering is an important parameter since it can reduce the sharpness of the interfaces.

### Surface Roughness Measurements

**Figure 3:**
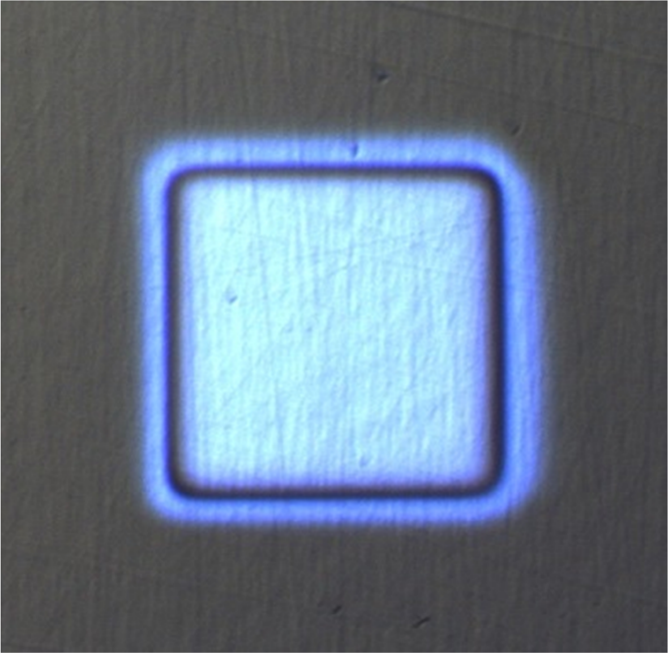
Crater caused by etching the CrTiAl sample with the 1 keV Cs^+^ ion beam.

The craters where the surface roughnesses were measured were ion sputtered in the SIMS instrument with a 1 or 2 keV Cs^+^ ion beam. An example of an optical image of such a crater is shown in Figure 3. Two craters were sputtered in UHV and two in a H_2_ atmosphere of 7 × 10^–7^ mbar. During the crater sputtering a Bi^+^ analysis ion beam was used as well, and we were able to see the multilayer structure that we were profiling. Following the SIMS signal evolution, we were therefore always able to stop the ion sputtering at the desired depth, either in the middle of the layer or exactly at the interface. In such a way the differences in the ion current, as well as sputtering rate, were considered, as slightly different times were needed to reach the desired depth while ion sputtering the same sample. The AFM analyses were performed in ambient conditions after all four craters were sputtered. Two 5 μm × 5 μm images were measured in each of the craters, so overall four images for the UHV conditions and four for the H_2_ atmosphere ion sputtering were made. The 2 μm × 2 μm images were recorded in the same manner. Analyses of the nonsputtered surface were made a few millimeters from the craters so that long-range differences did not affect the results. The analyses made too close to the crater are problematic as the debris originating from the ion sputtering could affect the measurements. The 2 μm × 2 μm areas were always analyzed inside the 5 μm × 5 μm areas, which were analyzed first.

**Figure 4:**
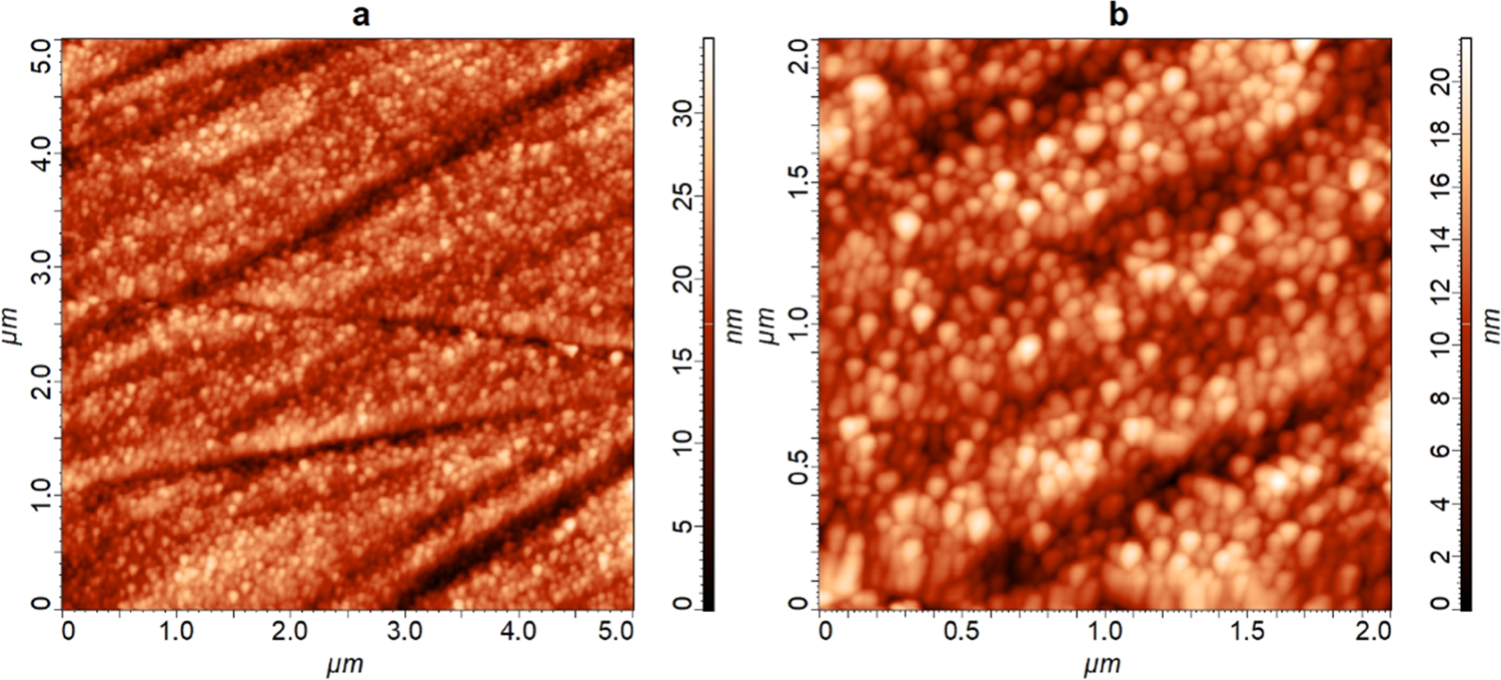
AFM images of the NiO layer at a depth of around 100 nm in the FeAgNi sample recorded inside the depth-profiling crater; 1 keV Cs^+^ ions were used for sputtering in the H_2_ atmosphere. Image (a) was taken over the 5 μm × 5 μm analysis area, while image (b) was measured inside the boundaries of this area over 2 μm × 2 μm while choosing the area with the least amount of large structural defects, such as ripples and ridges (seen in image (a)).

AFM measurements showed that the FeAgNi, CrTiAl, and TiSi samples all have relatively large initial surface roughnesses. In the case of the FeAgNi and CrTiAl samples, it was 5 ± 1 nm, and in the case of the TiSi sample, it was 3.4 ± 0.5 nm for the 5 μm × 5 μm analysis area. When the 2 μm × 2 μm area was analyzed, the surface roughness values were slightly lower: 3.5 ± 0.5 nm for the FeAgNi, 3.4 ± 0.4 nm for the CrTiAl, and 2.1 ± 0.4 nm for the TiSi sample. Figure 4 shows AFM images of the NiO layer in the FeAgNi sample recorded over the 5 μm × 5 μm and 2 μm × 2 μm analyses areas inside a crater after depth profiling with the 1 keV Cs^+^ ion beam in the H_2_ atmosphere.

**Figure 5:**
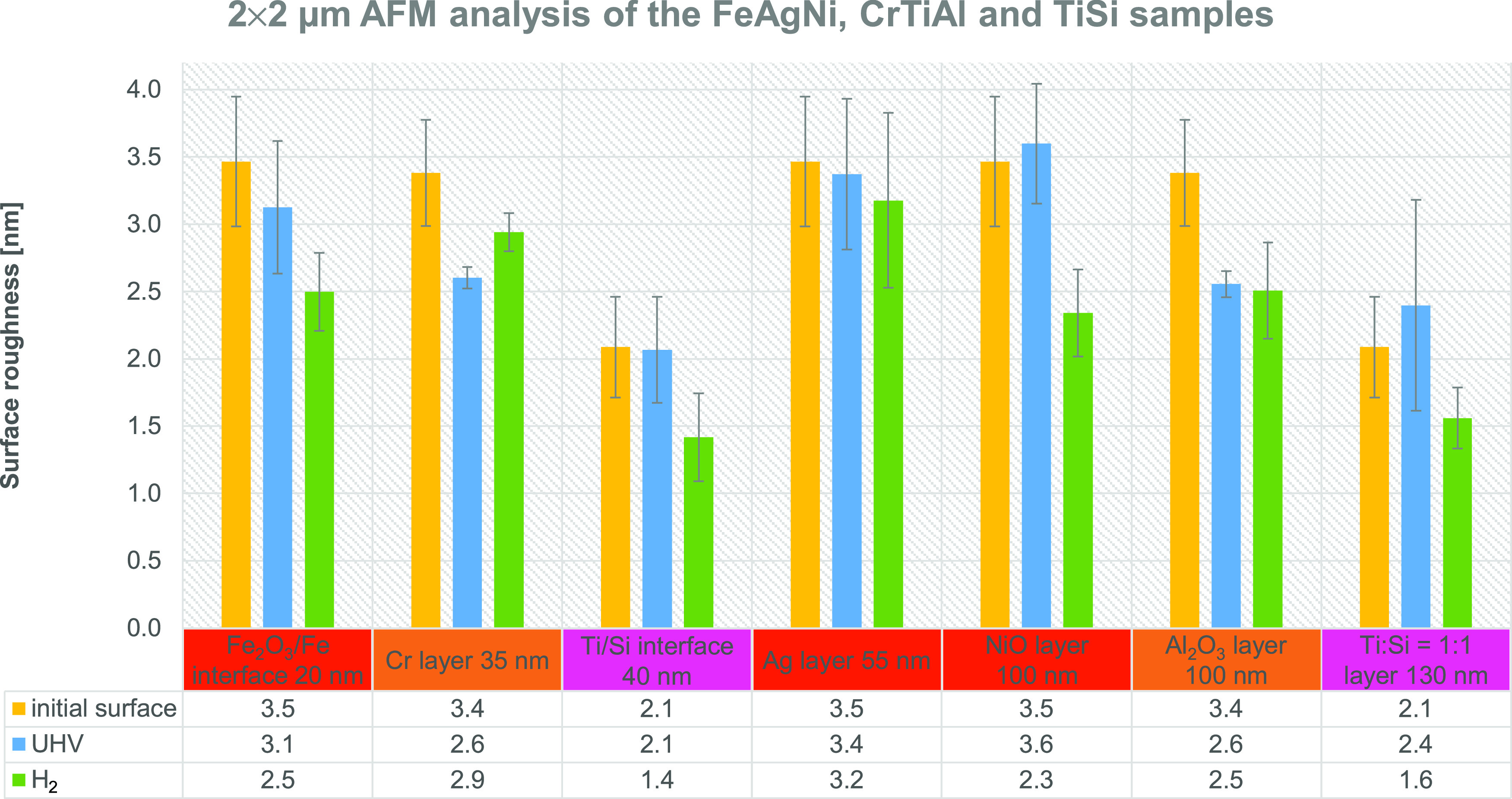
Surface roughness of the FeAgNi, CrTiAl, and TiSi samples with a table of the average surface roughness values. Surface roughness was measured over the 2 μm × 2 μm area. Yellow columns represent the initial surface roughness of the chosen sample, blue columns are the roughness of the craters sputtered in the UHV conditions, and green columns are the roughness after sputtering in the H_2_ atmosphere. Sputtering was made with the 1 keV Cs^+^ ion beam. The layers and interfaces measured on the FeAgNi sample are colored red, the ones from the CrTiAl sample are orange, and the ones from the TiSi sample are pink.

Figure 5 graphically presents the initial surface roughness of the first three samples (FeAgNi, CrTiAl, and TiSi) together with the surface roughness measured in the layers and interfaces noted previously after sputtering with the 1 keV Cs^+^ ion beam in the UHV conditions and during H_2_ flooding. The initial surface roughness is presented beside each layer and interface, with the sputtered depth increasing from left to right. The data presented in Figure 5 were measured over the 2 μm × 2 μm analysis area. We also added a table of the average surface roughness values below the graph to present our findings more clearly. The average surface roughness values for the 5 μm × 5 μm analysis area are between 3.1 ± 0.4 and 5.1 ± 0.5 nm and for the area of 2 μm × 2 μm, between 1.4 ± 0.3 and 3.6 ± 0.4 nm. All of the surface roughness values with standard deviations are listed in Supporting Information Tables S1–S3 for the FeAgNi, CrTiAl, and TiSi samples, respectively. A pronounced surfaced roughness is correlated with high standard deviation values and therefore many changes noticed during the depth profiling cannot be regarded as statistically significant. Nevertheless, we can still find and emphasize a few trends that are also statistically significant:

- Sputtering with the 1 keV Cs^+^ ion beam in the UHV environment or H_2_ atmosphere either does not change the initial surface roughness significantly (Ti/Si interface, Ag and Ti/Si = 1:1 layers) or can reduce it (Cr and Al_2_O_3_ layers, Fe_2_O_3_/Fe interface and NiO layer in the H_2_ atmosphere).
- Surface roughness can be reduced when the ion sputtering is performed in a H_2_ atmosphere compared to UHV conditions (significantly for the NiO layer and probably for the Ti/Si interface as well). Sputtering in UHV produces lower values of roughness than sputtering in H_2_ only for the Cr layer.
- Surface roughness is less affected by the sputtering depth than by the chemical composition of the layer or the interface of interest. The differences are within the statistically accepted uncertainty; however, the average surface roughness values for the FeAgNi sample, as an example, sputtered in the H_2_ atmosphere follow the trend of an initial decrease (Fe_2_O_3_/Fe interface), then an increase (Ag layer), and another decrease (NiO layer).

The surface roughness changes measured over the 5 μm × 5 μm area are shown in Figure S1. All of the main characteristics and trends are the same as for the 2 μm × 2 μm analysis area. There are only slightly changed average values for the differences between the surface roughness for the nonsputtered surface, the one sputtered in the UHV, and the one in the H_2_ atmosphere. Small differences can also be seen when comparing the standard deviation values between the graphs for the 5 μm × 5 μm and 2 μm × 2 μm analyses areas.

**Figure 6:**
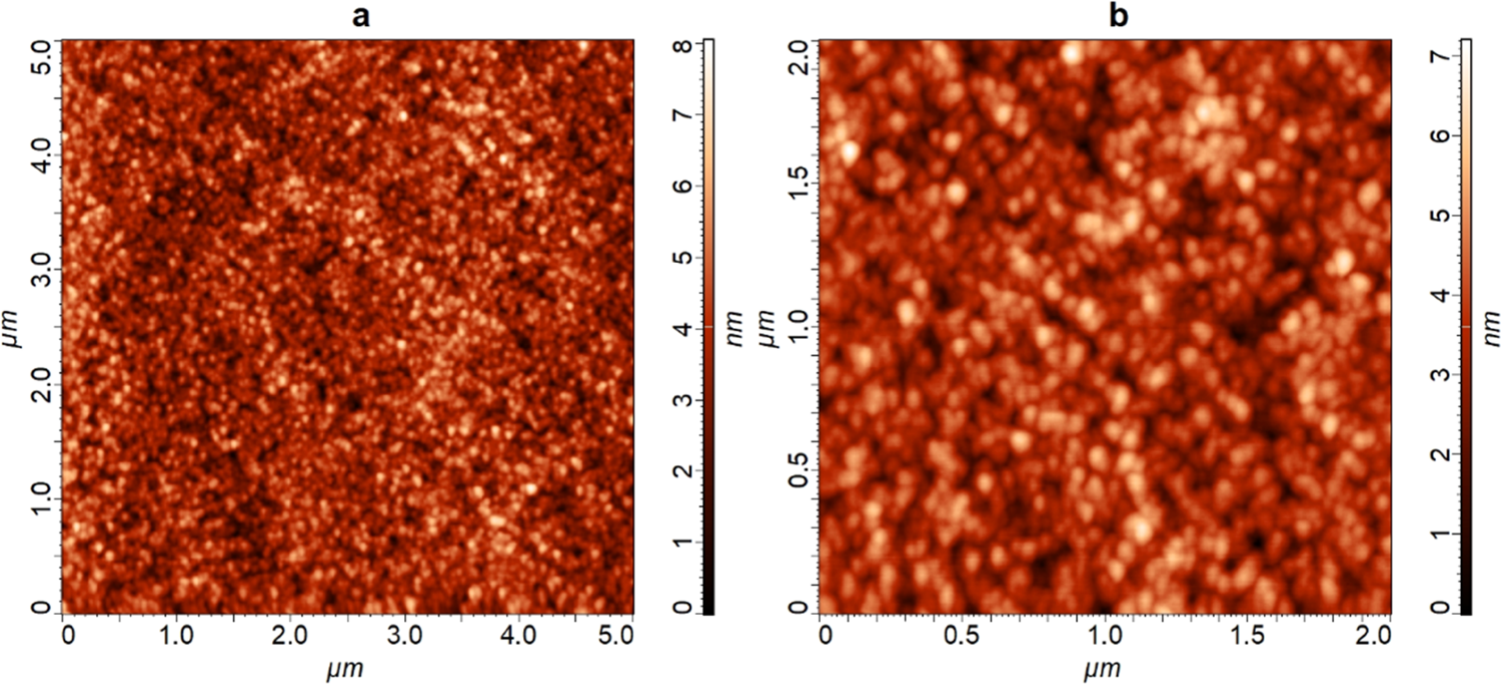
AFM images of the third Ni layer at a depth of around 135 nm in the NiCr sample recorded inside the depth-profiling crater; 1 keV Cs^+^ ions were used for sputtering in the H_2_ atmosphere. Image (a) was taken over a 5 μm × 5 μm analysis area, while image (b) was measured inside the boundaries of this area over 2 μm × 2 μm.

The NiCr sample is, on the other hand, much smoother than the FeAgNi, CrTiAl, and TiSi samples. Its initial surface roughnesses are 0.9 ± 0.2 and 0.6 ± 0.1 nm for the 5 μm × 5 μm and 2 μm × 2 μm analyses areas, respectively. This can also be observed on the AFM images as there are no lines caused by the sample preparation. Such ripples are, on the other hand, present in the cases of the FeAgNi (Figure 4), CrTiAl, and TiSi samples. Figure 6 shows AFM images of the third Ni layer in the NiCr sample recorded over the 5 μm × 5 μm and 2 μm × 2 μm analyses areas after sputtering. The depth profiling was, as in Figure 4, performed with the 1 keV Cs^+^ ion beam in the H_2_ atmosphere.

**Figure 7:**
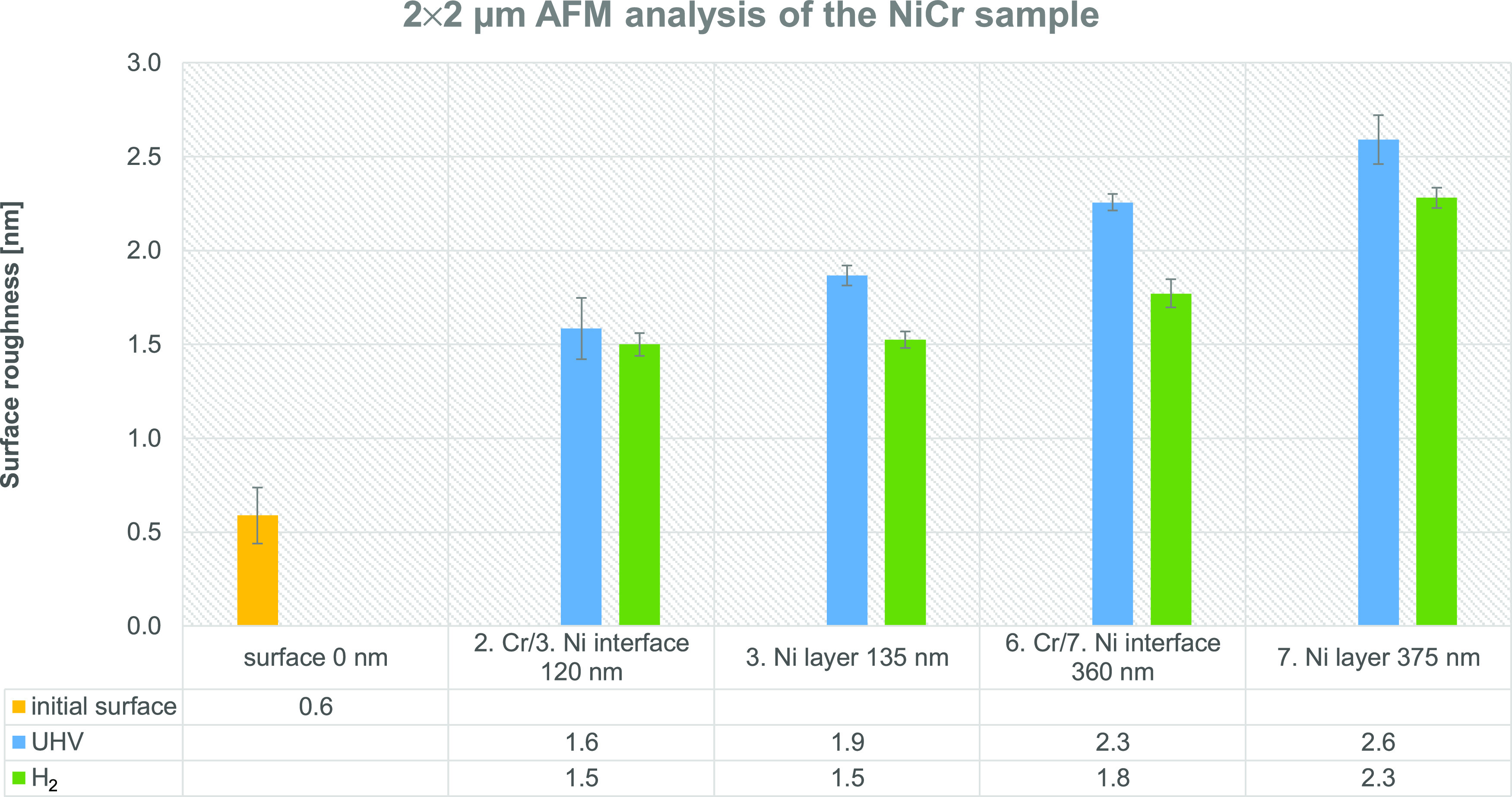
Surface roughness of the NiCr sample with a table of the average surface roughness values. Surface roughness was measured over the 2 μm × 2 μm area. Yellow column represents the initial surface roughness, blue columns the roughness of the craters sputtered in the UHV conditions, and green columns the roughness after sputtering in the H_2_ atmosphere. Sputtering was made with the 2 keV Cs^+^ ion beam.

Figure 7 presents the surface roughness of the NiCr sample measured over the 2 μm × 2 μm area. The roughness was measured on a nonsputtered surface and in the craters, etched with the 2 keV Cs^+^ ion beam, at depths of approximately 120, 135, 360, and 375 nm. The layers and interfaces were again etched in the UHV and during H_2_ flooding, with the results compared in Figure 7. The average surface roughness values with their standard deviations for both 5 μm × 5 μm and 2 μm × 2 μm analyses areas are listed in Table S4. The result is that the *S*_a_ measured over the 5 μm × 5 μm area increases from 0.9 ± 0.2 nm at the surface to 2.66 ± 0.05 nm (UHV) or 2.50 ± 0.05 nm (H_2_) at a depth of 375 nm. The change of *S*_a_ observed over the 2 μm × 2 μm area is from 0.6 ± 0.1 to 2.6 ± 0.1 nm (UHV) or 2.28 ± 0.05 nm (H_2_). The consequence of the much smoother surface of the NiCr sample is also a significantly lower standard deviation of the surface roughness compared to the FeAgNi, CrTiAl, and TiSi samples. Statistically significant conclusions are therefore easier to draw and more clearly pronounced:

- Surface roughness increases with a prolonged sputter time, and therefore with the depth of the crater being sputtered.
- H_2_ flooding notably reduces the surface roughening caused by the Cs^+^ ion sputtering.
- The effect of the H_2_ atmosphere becomes more evident at greater depths, and therefore after a longer sputter time.

The first observation was, however, expected since it is well known that ion sputtering with small projectiles (mainly monoatomic ions) increases surface roughening. Therefore, the results regarding surface roughness changes during ion sputtering presented in Figure 5 are worthy of more attention, since “polishing” of the surface with the Cs^+^ ion beam is not a well-known effect. Also, the surface roughness for the NiCr sample measured over the 5 μm × 5 μm analysis area shows the same pattern as the roughness measured over the 2 μm × 2 μm area. The graph showing the results from the 5 μm × 5 μm AFM analysis is shown in Figure S2.

**Figure 8:**
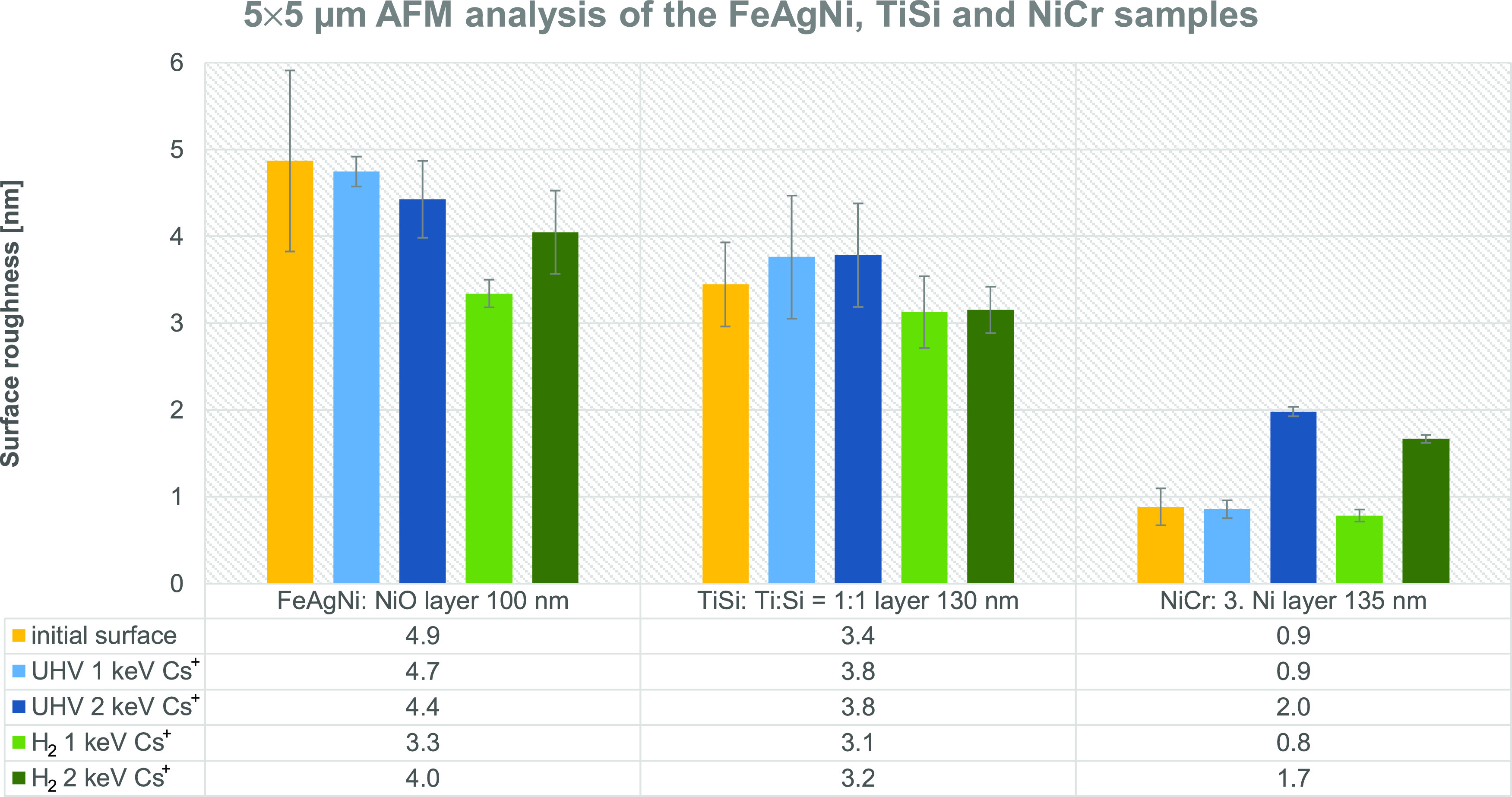
Surface roughness of the FeAgNi, TiSi, and NiCr samples after sputtering with the 1 and 2 keV Cs^+^ ion beams. Added is the table of average surface roughness values. Surface roughness was measured over the 5 μm × 5 μm area. Yellow columns represent the initial surface roughness, light blue columns the roughness of the craters sputtered in UHV with the 1 keV Cs^+^, dark blue columns the roughness of the craters sputtered in UHV with the 2 keV Cs^+^, light green columns the roughness after sputtering with the 1 keV Cs^+^ in the H_2_ atmosphere, and dark green columns the roughness after sputtering with the 2 keV Cs^+^ in the H_2_ atmosphere. Analyses of the layers are assigned as “the sample: the layer of the sample being analyzed and the depth at which ion sputtering was stopped”.

Finally, we compared the surface roughening caused by Cs^+^ ion beams with different energies, i.e., 1 and 2 keV. We must also emphasize that we tested only the Cs^+^ ion beam since our previous study^12^ showed that H_2_ flooding works optimally when combined with Cs^+^ sputtering. Since the main objective of this study is to show the effects of the H_2_ atmosphere on surface roughening during depth profiling, we did not include other types of sputtering ions. Figure 8 shows a graph comparing the surface roughness of the specific layers of the FeAgNi, TiSi, and NiCr samples after etching with both the 1 and 2 keV Cs^+^ ion beams in the UHV and H_2_ atmosphere. The AFM analysis for Figure 8 was made over the 5 μm × 5 μm area. The results for the 2 μm × 2 μm analysis area shown in Figure S3 reveal the same trends as the 5 μm × 5 μm AFM analysis. The average surface roughness values and their standard deviations used in Figures 8 and S3 are listed in Tables S1–S4. The results shown in Figure 8 confirm the findings from Figures 5 and 7, as well as offer some additional information:

- The 2 keV Cs^+^ ion beam causes a more pronounced roughening than the 1 keV Cs^+^ beam if the surface initially has a low surface roughness (NiCr sample). If the surface is initially rougher (FeAgNi and TiSi samples), then there is no statistical difference when sputtering with Cs^+^ ions of different energies.
- The 1 keV Cs^+^ sputtering either does not cause any significant change in the surface roughness (TiSi sample) or it can, for some layers and during H_2_ flooding, reduce the roughness (FeAgNi and CrTiAl samples).
- H_2_ flooding either reduces the roughening compared to the UHV conditions or does not affect the surface roughening. The positive H_2_ effect is the most clearly seen for the NiO layer (FeAgNi sample) when sputtering with the 1 keV Cs^+^ and for the third Ni layer (NiCr sample) when sputtering with the 2 keV Cs^+^ ion beam.

### Discussion

From the surface roughness measurements, we can conclude that the H_2_ flooding during SIMS depth profiling potentially leads to reduced surface roughening caused by the ion sputtering. This can be clearly seen in Figures 5, 7, 8, and S1–S3. Namely, almost all of the AFM analyses of the layers and interfaces show reduced average surface roughness values when H_2_ flooding was applied, compared to the UHV environment. The reduction in the surface roughness is sometimes statistically significant, while in other cases it cannot be confirmed. The only exception is the Cr layer in the CrTiAl sample (Figures 5 and S1), where the surface roughness during H_2_ flooding increases compared to the UHV depth profiling. Such observations are positive, as they indicate that the H_2_ atmosphere applied during the depth profiling of metals, metal oxides, and alloys not only improves the capability of the SIMS method to unambiguously distinguish different layers^12^ but also potentially reduces the surface roughening caused by the ion-induced damage. The results are even better when we put them into the perspective of the sputter rate, which does not change significantly when the H_2_ atmosphere and UHV are compared.^12^ Namely, we anticipate that a very thin surface layer of metal hydride is formed during H_2_ flooding, which also causes hydrogen-induced embrittlement of the metals.^50−52^ The embrittlement explains the unchanged sputter rate, which is otherwise reduced if gases such as O_2_, CO, or C_2_H_2_ are applied instead of the H_2_.^12^

Another important observation is connected with the initial surface roughness of our samples. Namely, if the sample is very flat (NiCr sample with a surface roughness below 1 nm), ion sputtering either leads to a gradual surface roughening (2 keV Cs^+^ ion beam, Figures 7, 8, S2, and S3) or the surface roughness remains more or less unchanged (1 keV Cs^+^ ion beam, Figures 8 and S3). Such results are expected since the surface roughening caused by the ion-bombardment-induced damage is a well-known phenomenon.^20−22^ The roughness of the surfaces increases during the ion sputtering due to the formation of different topographical structures such as ripples, ridges, and cones/pyramids.^35,40^ It is, therefore, unexpected that especially the 1 keV Cs^+^ ion beam offers the potential for smoothing the surface of a sample that is not very flat (surface roughness above 3 nm). Namely, as seen in Figures 5 and S1, the surface roughness of the specific layers of the FeAgNi and CrTiAl samples, which have a greater initial surface roughness, decreases after they are sputtered with the 1 keV Cs^+^ ion beam, either during H_2_ flooding and/or in the UHV environment. This is most clearly seen for the Fe_2_O_3_/Fe interface and the Cr, NiO, and Al_2_O_3_ layers. In these cases, we can observe a statistically significant decrease of the surface roughness in at least one of the measurement areas (2 μm × 2 μm or 5 μm × 5 μm) and during the depth profiling in at least one of the environments (H_2_ atmosphere or UHV). The 1 keV Cs^+^ depth profiling is therefore very suitable when surface roughening during a depth profiling is highly undesirable, as well as when polishing of the initially rough surface is desired. However, we must also emphasize that this is not the only example of surface smoothing achieved with ion sputtering. For example, a bombardment of a SiO_2_ surface with an initial roughness of approximately 1 nm with the 0.2–1.0 keV H^+^ ions resulted in a reduced surface roughness.^53^

As already mentioned, greater surface roughening can be seen when a Cs^+^ ion beam of higher energy is used (Figures 8 and S3). Such results are expected since ions of higher energy cause greater damage to the surface during their impact and similar findings were already published.^21,32,33^ We must, however, note that an increase in the surface roughening is not always correlated with an increase in the energy of the sputtering ions. O_2_^+^ sputtering is such an example, as the onset of the surface roughening during this process happens, in some specific cases defined by the incident angle of the ion beam, sooner and also to a greater extent if O_2_^+^ ions of lower energy are used.^54,55^ We can, therefore, conclude that the results of our study can be applied only as a confirmation of the correlation between the surface roughening and the energy of the Cs^+^ ion beam, but cannot be extended to the other types of sputter ions.

Our study, furthermore, shows a correlation between the sputtering depth (or the sputter time) and the surface roughness, but only in the case of an initially flat NiCr sample. As seen in Figures 7 and S2, the surface roughness of the NiCr sample increases with the increasing sputter depth reached during sputtering with the 2 keV Cs^+^ ion beam, both when H_2_ flooding or UHV conditions were applied. Such results are expected, as previous studies show the same correlation.^21,33−35^ However, different results were found for the initially rougher FeAgNi, CrTiAl, and TiSi samples, where no correlation between the surface roughness and the sputtering depth can be determined when sputtering with 1 keV Cs^+^ ions (Figures 5 and S1). We believe that different effects are behind such observations. Namely, as we already determined, sputtering with a 1 keV Cs^+^ ion beam can reduce the surface roughness of initially rough samples. Therefore, increased roughening should not be expected. On the other hand, we also cannot observe any continuous decrease of the surface roughness, but rather random decreases and increases, which are in many cases statistically insignificant. As such, a layer that was lying deeper in the sample and needed a longer sputtering time could have a smaller surface roughness than the layer above it, or the other way around. These decreases and increases in the surface roughness are, however, correlated for the 2 μm × 2 μm and 5 μm × 5 μm analyses areas when the same layer and the same profiling condition (H_2_ atmosphere or UHV) are compared. The most probable explanation for this is the effect of the chemical composition and crystallographic structure of the layer. Namely, different layers can have different initial crystallinity and also exhibit different tendencies to form an amorphous layer on the surface as a consequence of the ion sputtering. It was already proven that many materials amorphize during ion sputtering.^32,34,56−58^ Amorphization affects the surface roughness, generally reducing it as amorphous materials more easily relax and fewer topographical structures are formed.^34^ Furthermore, hydrides and hydroxides, formed during depth profiling in the H_2_ atmosphere, and mostly oxides, formed in the UHV, can exhibit different roughnesses depending on the metal they bind with. Mostly oxides are formed in UHV because some O_2_ is still present, while during H_2_ flooding, we observe the formation of hydrides when metals are being sputtered, and the formation of hydroxides when sputtering metal oxide layers. Flooding of the oxygen during depth profiling as well as the formation of the oxides in the profiling crater both reduce the surface roughening.^38,39^ We believe that a similar effect can be observed for metallic hydrides and hydroxides.

## Conclusions

The results of our study show that H_2_ flooding applied during SIMS depth profiling with a Cs^+^ ion beam positively affects many aspects of the measurements. Besides the improved resolving capability of SIMS due to the reduced matrix effect, also a reduced surface roughening can be observed in comparison to the UHV environment. Namely, the surface roughness of different metallic and metal oxide layers measured during AFM was lower when they were depth-profiled in the H_2_ atmosphere instead of the UHV or there was no statistical difference. Only one exception was observed. Furthermore, our research posts another proof for the ion-energy-dependent roughening, as we have shown that the Cs^+^ ion beam with an energy of 2 keV causes more damage and roughening than the 1 keV Cs^+^ ion beam. We even observed smoothing capabilities with the 1 keV Cs^+^ ions. If the surface of our samples was initially rough (more than 3 nm), sputtering with Cs^+^ ions having an energy of 1 keV led either to a reduction of surface roughness in cases of some layers in the FeAgNi and CrTiAl samples, or there was no statistically significant difference before and after sputtering. Another important observation indicates that surface roughness is also dependent on the chemical composition of the layer, as a different surface roughness was measured for chemically different layers of the same sample regardless of their sputter depth. Since the only unchanged parameter was the chemical composition of the layers, we believe that the formation of hydrides, hydroxides, and oxides with different crystallinity is the reason for the different surface roughness values. Namely, a different degree of order in the structure of the material, i.e., the material being more crystalline or amorphous, can cause different surface roughnesses. Amorphization can also be caused by ion sputtering since different materials are differently prone to form amorphous phases. On the other hand, when the surface roughnesses of layers of the same chemical composition were measured through different depths (NiCr sample), their roughness continuously and gradually increased with increasing sputter depth. Such a result was expected since prolonged sputtering also leads to the accumulation of ion-bombardment-induced damage. Future studies will be performed to find the relationships between the surface morphology and the depth resolution, i.e., broadening of the interfaces observed in the ToF-SIMS depth profiles.

## Supporting Information Available

Additional graphs presenting surface roughness measured over the 2 μm × 2 μm or 5 μm × 5 μm analyses areas and tables of average surface roughness values with their standard deviations (PDF)
