# Supplementary material for: AFM Study of Roughness Development during ToF-SIMS Depth Profiling of Multilayers with a Cs+ Ion Beam in a H2 Atmosphere
Source: Langmuir. 2022 Oct 14;38(42):12871–80. doi: 10.1021/acs.langmuir.2c01837 (PMC9609309; doi:10.1021/acs.langmuir.2c01837)
Supplement: Supplementary file 1 — la2c01837_si_001.pdf [file la2c01837_si_001.pdf]

Supporting information

AFM Study of Roughness Development during ToF-SIMS Depth Profiling of Multilayers with a Cs^+ Ion Beam in a H_2 Atmosphere

Jernej Ekar,[1,2] *Janez Kovač*[1,*]

[1]Jožef Stefan Institute, Jamova cesta 39, SI-1000 Ljubljana, Slovenia

[2]Jožef Stefan International Postgraduate School, Jamova cesta 39, SI-1000 Ljubljana, Slovenia

Corresponding author: * Janez Kovač (email: janez.kovac@ijs.si, phone: 00386 1 477 34 03)

Table S1. Measured surface roughness S_a values in nm with their standard deviations for the layers of the FeAgNi sample.

FeAgNi sample		Roughness S_a over 5 × 5 μm^2		Roughness S_a over 2 × 2 μm^2	
		1 keV Cs^+	2 keV Cs^+	1 keV Cs^+	2 keV Cs^+
initial surface roughness*		5 ± 1		3.5 ± 0.5	
Fe_2O_3/Fe interface at 20 nm	UHV	4.6 ± 0.6	/	3.1 ± 0.5	/
	H_2	4.3 ± 0.7	/	2.5 ± 0.3	/
Ag layer at 55 nm	UHV	5.1 ± 0.5	/	3.4 ± 0.6	/
	H_2	4.3 ± 0.6	/	3.2 ± 0.6	/
NiO layer at 100 nm	UHV	4.7 ± 0.2	4.4 ± 0.4	3.6 ± 0.4	3.1 ± 0.6
	H_2	3.3 ± 0.2	4.0 ± 0.5	2.3 ± 0.3	3.0 ± 0.5

Measured surface roughness values are for the 5 μm × 5 μm and 2 μm × 2 μm areas, UHV conditions and H_2 flooding, as well as for the 1 and 2 keV energies of the Cs^+ sputtering ions.

* Initial surface roughness values correspond only to the 5 μm × 5 μm and 2 μm × 2 μm analysis areas since no prior sputtering was done in cases of these AFM measurements.

Table S2. Measured surface roughness S_a values in nm with their standard deviations for the layers of the CrTiAl sample.

CrTiAl sample		Roughness S_a over 5 × 5 μm^2		Roughness S_a over 2 × 2 μm^2	
		1 keV Cs^+	2 keV Cs^+	1 keV Cs^+	2 keV Cs^+
initial surface roughness*		5 ± 1		3.4 ± 0.4	
Cr layer at 35 nm	UHV	3.4 ± 0.3	/	2.60 ± 0.08	/
	H_2	4.3 ± 0.2	/	2.9 ± 0.1	/
Al_2O_3 layer at 100 nm	UHV	4.6 ± 0.4	/	2.6 ± 0.1	/
	H_2	4.0 ± 0.4	/	2.5 ± 0.4	/

Measured surface roughness values are for the 5 μm × 5 μm and 2 μm × 2 μm areas, UHV conditions and H_2 flooding, measured after sputtering with the 1 keV Cs^+ ions.

* Initial surface roughness values correspond only to the 5 μm × 5 μm and 2 μm × 2 μm analysis areas since no prior sputtering was done in cases of these AFM measurements.

Table S3. Measured surface roughness S_a values in nm with their standard deviations for the layers of the TiSi sample.

TiSi sample		Roughness S_a over 5 × 5 μm^2		Roughness S_a over 2 × 2 μm^2	
		1 keV Cs^+	2 keV Cs^+	1 keV Cs^+	2 keV Cs^+
initial surface roughness*		3.4 ± 0.5		2.1 ± 0.4	
Ti/Si interface at 40 nm	UHV	4.1 ± 0.8	/	2.1 ± 0.4	/
	H_2	3.3 ± 0.9	/	1.4 ± 0.3	/
Ti:Si = 1:1 layer at 130 nm	UHV	3.8 ± 0.7	3.8 ± 0.6	2.4 ± 0.8	2.2 ± 0.4
	H_2	3.1 ± 0.4	3.2 ± 0.3	1.6 ± 0.2	2.0 ± 0.4

Measured surface roughness values are for the 5 μm × 5 μm and 2 μm × 2 μm areas, UHV conditions and H_2 flooding, as well as for the 1 and 2 keV energies of the Cs^+ sputtering ions.

* Initial surface roughness values correspond only to the 5 μm × 5 μm and 2 μm × 2 μm analysis areas since no prior sputtering was done in cases of these AFM measurements.

Table S4. Measured surface roughness S_a values in nm with their standard deviations for the layers of the NiCr sample.

NiCr sample		Roughness S_a over 5 × 5 μm^2		Roughness S_a over 2 × 2 μm^2	
		1 keV Cs^+	2 keV Cs^+	1 keV Cs^+	2 keV Cs^+
initial surface roughness*		0.9 ± 0.2		0.6 ± 0.1	
2. Cr/3. Ni interface at 120 nm	UHV	/	1.6 ± 0.2	/	1.6 ± 0.2
	H_2	/	1.61 ± 0.05	/	1.50 ± 0.06
3. Ni layer at 135 nm	UHV	0.9 ± 0.1	1.98 ± 0.06	0.8 ± 0.1	1.87 ± 0.05
	H_2	0.79 ± 0.07	1.67 ± 0.05	0.69 ± 0.08	1.52 ± 0.05

6. Cr/7. Ni interface at 360 nm	UHV	/	2.41 ± 0.06	/	2.26 ± 0.04
	H_2	/	2.07 ± 0.02	/	1.77 ± 0.08
7. Ni layer at 375 nm	UHV	/	2.66 ± 0.05	/	2.6 ± 0.1
	H_2	/	2.50 ± 0.05	/	2.28 ± 0.05

Measured surface roughness values are for the 5 µm × 5 µm and 2 µm × 2 µm areas, UHV conditions and H_2 flooding, as well as for the 1 and 2 keV energies of the Cs^+ sputtering ions.

* Initial surface roughness values correspond only to the 5 µm × 5 µm and 2 µm × 2 µm analysis areas since no prior sputtering was done in cases of these AFM measurements.

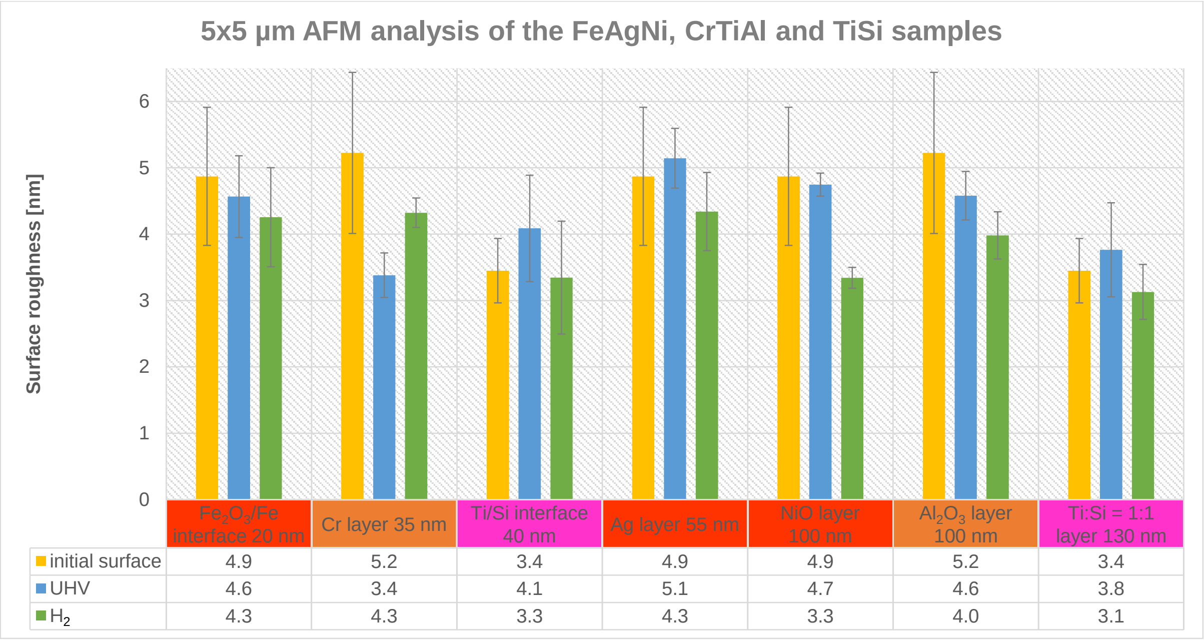

Figure S1. Surface roughness of the FeAgNi, CrTiAl, and TiSi samples with a table of the average surface roughness values. Surface roughness was measured over the 5 µm × 5 µm area. Yellow columns represent the initial surface roughness of the chosen sample, blue columns are the roughness of the craters sputtered in the UHV conditions, and green columns are the roughness after sputtering in the H_2 atmosphere. Sputtering was made with the 1 keV Cs^+ ion beam. The layers and interfaces measured on the FeAgNi sample are colored red, the ones from the CrTiAl sample are orange, and the ones from the TiSi sample are pink.

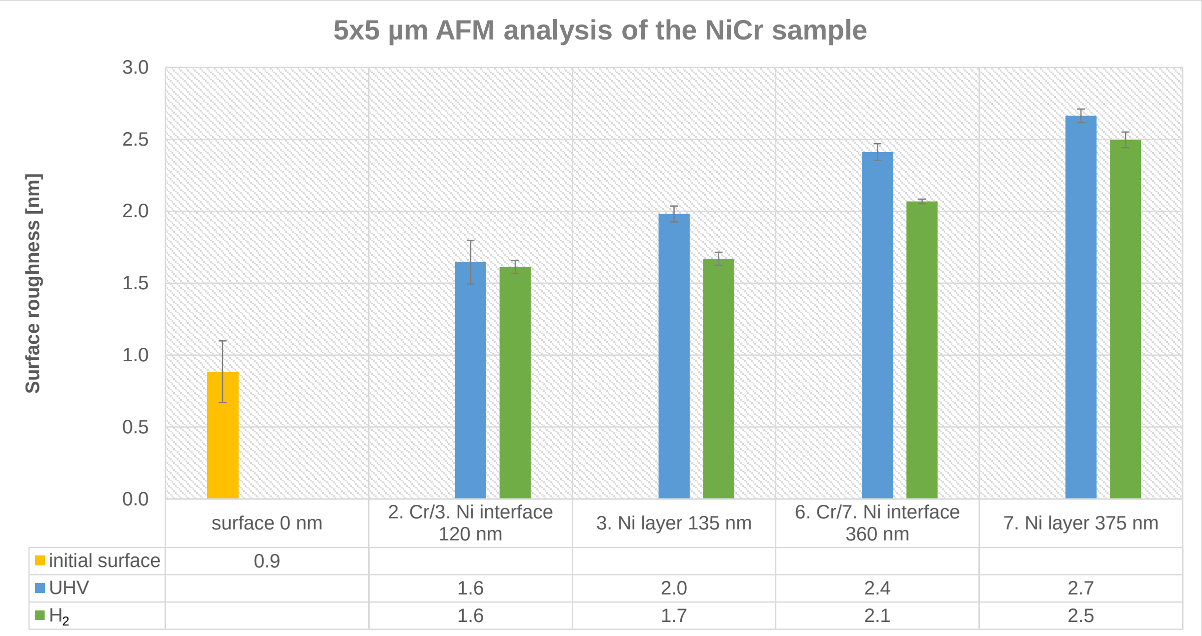

	surface 0 nm	2. Cr/3. Ni interface 120 nm	3. Ni layer 135 nm	6. Cr/7. Ni interface 360 nm	7. Ni layer 375 nm
initial surface	0.9				
UHV		1.6	2.0	2.4	2.7
H_2		1.6	1.7	2.1	2.5

Figure S2. Surface roughness of the NiCr sample with a table of the average surface roughness values. Surface roughness was measured over the 5 µm × 5 µm area. Yellow column represents the initial surface roughness, blue columns the roughness of the craters sputtered in the UHV conditions, and green columns the roughness after sputtering in the H_2 atmosphere. Sputtering was made with the 2 keV Cs^+ ion beam.

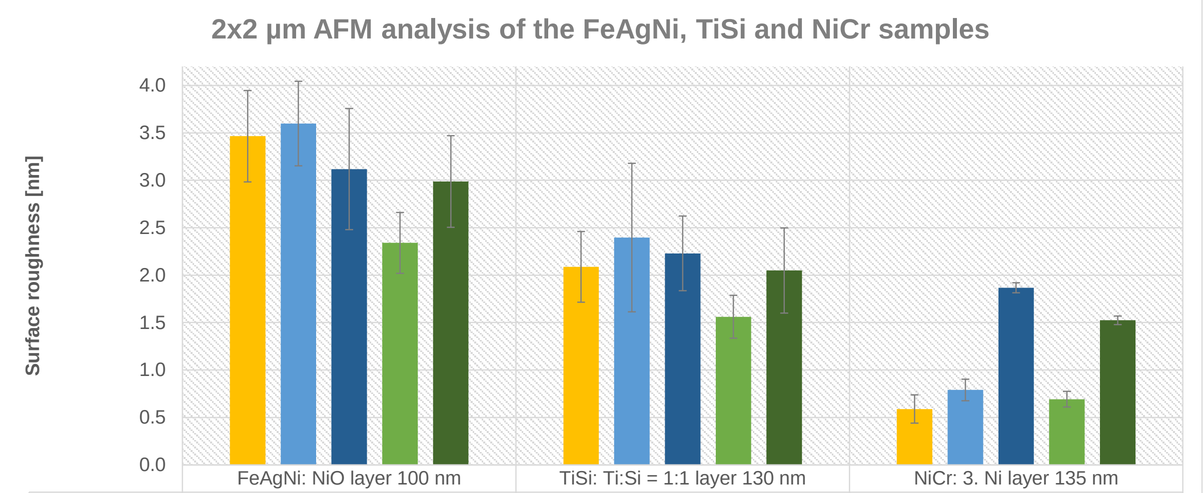

	FeAgNi: NiO layer 100 nm	TiSi: Ti:Si = 1:1 layer 130 nm	NiCr: 3. Ni layer 135 nm
initial surface	3.5	2.1	0.6
UHV 1 keV Cs^+	3.6	2.4	0.8
UHV 2 keV Cs^+	3.1	2.2	1.9
H_2 1 keV Cs^+	2.3	1.6	0.7
H_2 2 keV Cs^+	3.0	2.0	1.5

Figure S3. Surface roughness of the FeAgNi, TiSi, and NiCr samples after sputtering with the 1 and 2 keV Cs^+ ion beams. Added is the table of average surface roughness values. Surface roughness was measured over the 2 µm × 2 µm area. Yellow columns represent the initial surface roughness, light blue columns the roughness of the craters sputtered in UHV with the 1 keV Cs^+, dark blue columns the roughness of the craters sputtered in UHV with the 2 keV Cs^+, light green columns the roughness after sputtering with the 1 keV Cs^+ in the H_2 atmosphere, and dark green columns the roughness after sputtering with the 2 keV Cs^+ in the H_2 atmosphere. Analyses of the layers are assigned as “the sample: the layer of the sample being analyzed and the depth at which ion sputtering was stopped”.
